# Arcuate AgRP neurons and the regulation of energy balance

**DOI:** 10.3389/fendo.2012.00169

**Published:** 2012-12-27

**Authors:** Céline Cansell, Raphaël G. P. Denis, Aurélie Joly-Amado, Julien Castel, Serge Luquet

**Affiliations:** ^1^Unité de Biologie Fonctionnelle et Adaptative, CNRS-EAC 4413, Sorbonne Paris Cité, Université Paris Diderot-Paris 7Paris, France; ^2^Centre National de la Recherche Scientifique EAC 4413Paris, France

**Keywords:** neuropeptide Y, agouti-related protein, GABA, feeding behavior, metabolism, obesity, reward, dopamine

## Abstract

The arcuate nucleus of the hypothalamus contains at least two populations of neurons that continuously monitor signals reflecting energy status and promote the appropriate behavioral and metabolic responses to changes in energy demand. Activation of neurons making pro-opiomelanocortin (POMC) decreases food intake and increases energy expenditure through activation of G protein-coupled melanocortin receptors via the release of α-melanocyte-stimulating hormone. Until recently, the prevailing idea was that the neighboring neurons [agouti-related protein (AgRP) neurons] co-expressing the orexigenic neuropeptides, AgRP, and neuropeptide Y increase feeding by opposing the anorexigenic actions of the POMC neurons. However, it has now been demonstrated that only AgRP neurons activation – not POMC neurons inhibition – is necessary and sufficient to promote feeding. Projections of AgRP-expressing axons innervate mesolimbic, midbrain, and pontine structures where they regulate feeding and feeding-independent functions such as reward or peripheral nutrient partitioning. AgRP neurons also make gamma aminobutyric acid , which is now thought to mediate many of critical functions of these neurons in a melanocortin-independent manner and on a timescale compatible with neuromodulation.

During the last several decade, the world has witnessed a pandemic expansion of pathologies related to high-fat and high-carbohydrate diets including obesity, diabetes, dyslipidemia, and cardiovascular diseases – collectively referred to as metabolic syndrome. Obesity is now considered by the World Health Organization (WHO) to be a worldwide epidemic, having more than doubled since 1980. In 2008, there were 1.5 billion overweight adults in both developed and developing countries (http://www.who.int/mediacentre/factsheets/fs311/en/). The WHO believes the fundamental cause of obesity and being overweight is an energy imbalance between calories consumed and calories expended. Appropriate energy balance is reached when energy intake and energy expenditure are adapted to meet energy demands and nutrient availability. It took billions of years for mammalian species to shape a highly responsive homeostatic system in which the multiple aspects of energy expenditure are exquisitely balanced with both hunger and the motivational component of feeding to ensure energy homeostasis. Disruption of this regulation gives rise to life-threatening conditions that include anorexia nervosa at one extreme and metabolic syndrome at the other.

During the last decade, a significant effort has been focused on the identification of neuronal pathways that control food intake and energy expenditure. This review focuses primarily on a tiny neuronal population of about ~1000 cells located in arcuate nucleus (ARC) of the hypothalamus, namely the neurons that produce agouti-related protein (AgRP), neuropeptide Y (NPY), and gamma aminobutyric acid (GABA; referred to here as AgRP neurons), and the recent conceptual advances that have been made studying their function in energy balance.

## AgRP AND POMC NEURONS: TWO INTERMINGLED NEURONAL POPULATIONS DEFINING THE MELANOCORTIN SYSTEM

Agouti-related protein was discovered as an endogenously released neuropeptide that acts as an inverse agonist for the melanocortin receptors, MC3R/MC4R (Fan et al., 1997; Haskell-Luevano et al., 1999; Haskell-Luevano and Monck, 2001; Nijenhuis et al., 2001; Flier, 2006; Tolle and Low, 2008). Shortly after its discovery, Hahn et al. (1998) discovered that AgRP is co-expressed in hunger-activated neurons with NPY, another peptide that stimulates food intake and regulates weight gain (Tatemoto et al., 1982; Clark et al., 1984). The inhibitory nature of the NPY/AgRP neurons was further substantiated through the identification of GABA as their ionotropic neurotransmitter (Horvath et al., 1997). These neurons are now commonly referred to as AgRP neurons because, unlike NPY and GABA which are widely expressed in the nervous system, AgRP is uniquely produced by these neurons. It is a unique molecular signature that has been extensively exploited for the selective manipulation of these neurons. AgRP neurons are located in the ARC subdivision of the hypothalamus at the bottom of the third ventricle close to a circumventricular organ called median eminence (ME). The blood–brain barrier in this region is fenestrated and allows for facilitated blood–brain exchange. As a result, neurons that reside there are referred to as “first order neurons” because they would be the first to respond to the circulating signals of hunger and satiety.

**FIGURE 1:**
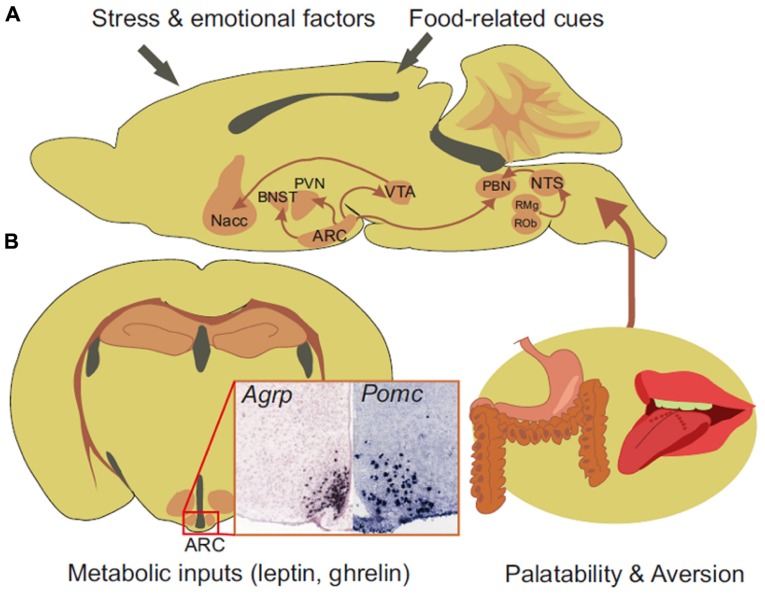
Sagittal **(A)** and coronal section **(B)** of a mouse brain showing *in situ* hybridization for mRNA encoding *Agrp* or *Pomc* and the connections recently described for AgRP neurons. Arcuate neurons project to the PVN, BNST, PBN, and VTA of the midbrain. The dopaminergic neurons of the VTA project to the nucleus accumbens (Nacc) to process the reward and motivational aspect of feeding. Gut-initiated viscerosensitive information and taste-related cues are routed to the NTS. The NTS targets the PBN and also receives serotoninergic input from the raphe obscurus (ROb) and the raphe magnus (RMg) and exerts an anorectic action through the glutamatergic excitation of the PBN. AgRP neurons integrate metabolic input of hunger and have direct connections to brain regions processing reward and motivation together with food-related cues such as palatability and aversive aspect. *In situ* hybridization picture were downloaded from the Allen Mouse Brain Atlas [http://mouse.brain-map.org/Seattle (WA): Allen Institute for Brain Science (Lein et al., 2007).© 2009].

Neurons in the ARC that make pro-opiomelanocortin (POMC) and cocaine- and amphetamine-related transcript (CART) secrete the melanocortin peptides adrenocorticotropic hormone (ACTH) and α, β, and γ-melanocyte-stimulating hormone (MSH), which are derived from post-translational processing of POMC. POMC and AgRP neurons are considered to be two functionally opposed components of the “central melanocortin system,” a term that refers to as a set of hormonal, neuropeptidergic, and paracrine signaling pathways that are defined by components that include the five G protein-coupled melanocortin receptors (MCR1 to MCR5; Cone, 2005). These receptors are distributed throughout the body (Mountjoy and Wong, 1997; Liu et al., 2003). In the CNS, MC4R is broadly expressed while MC3R is mainly restricted to POMC and AgRP neurons (Jegou et al., 2000). The integral role of the melanocortin system in body weight homeostasis is supported by the fact that any mutation in the melanocortin signaling pathway including MC3R- or MC4R-null mutants (Huszar et al., 1997) and ectopic expression of MCR3/4 antagonist, agouti, in agouti lethal yellow (A^y^) mutant mice (Miltenberger et al., 1997), results in hyperphagia, hypometabolism, hyperinsulinemia, and hyperglycemia in both rodents and humans (Hinney et al., 1999; Krude et al., 1999). The antagonistic relationship between POMC and AgRP neurons results from a tonic GABAergic inhibition from AgRP neurons onto POMC neurons (Horvath et al., 1992; Broberger and Hokfelt, 2001; Cowley et al., 2001; Williams et al., 2001; Pinto et al., 2004) and the interaction of NPY released by AgRP neurons with the NPY-Y1 receptor expressed on POMC neurons. The two populations project to several nuclei within the hypothalamus, including the paraventricular (PVN), ventromedial, dorsomedial, and lateral hypothalamus, and to the nucleus of tractus solitarii (NTS), the parabrachial nucleus (PBN) and the amygdala and the bed of the stria terminalis (BNST) which lie outside the hypothalamus (Broberger et al., 1998; **Figure 1**). In these regions AgRP- and NPY-containing fibers are found in close apposition to α-MSH-containing fibers and synapse onto second-order targets (Broberger et al., 1998). The release of α-MSH by POMC neurons and its binding to G-coupled MCR’s initiates the central anorexic signaling pathway that results in decreased food intake and increased energy expenditure while AgRP exerts its orexigenic action partly by blocking the binding of α-MSH to its receptor there by preventing the α-MSH-induced anorexic pathway (Michaud et al., 1994; Ollmann et al., 1997; Shutter et al., 1997; Haskell-Luevano et al., 1999; Haskell-Luevano and Monck, 2001; Nijenhuis et al., 2001; Tolle and Low, 2008).

Thyroid-releasing hormone (TRH)-, oxytocin (OT)-, and corticotropin-releasing hormone (CRH)-expressing neurons located in the PVN all express MC4R (Lu et al., 2003). The binding of α-MSH to MC4R on these neurons has a positive action onto the hypothalamic–pituitary–thyroid (HPT) axis and the hypothalamic–corticotropic axis (HPA). During fasting, increased release of AgRP by AgRP neurons has been demonstrated to be a key mechanism for fasting-induced down regulation of the HPT axis and the consequent adaptation during negative energy balance (Fekete et al., 2000; Lechan and Fekete, 2006).

These observations promoted a dominant conceptual framework that, until recently envisioned the orexigenic and anabolic action of AgRP neurons as the result of POMC neuron antagonism (Palmiter, 2012).

However, there are several lines of evidence supporting a melanocortin-independent pathway for AgRP. For exemple, short- and long-term hyperphagic actions of AgRP are still observed in MC4R KO mice and some AgRP fibers—but not α-MSH fibers—have been found in close apposition to TRH-synthesizing neurons expressing MC4R (Fekete et al., 2000). Moreover, it has been shown that AgRP can act by a melanocortin-independent pathway that regulates glutamatergic neurons in the ventromedial hypothalamus (Fu and van den Pol, 2008). These data were first to suggest that AgRP, can exert an action as an agonist on unidentified receptors that are independent from the melanocortin signaling pathway.

Recent studies expand further the role of AgRP neurons to include melanocortin-independent mechanisms and non-feeding-related functions such as goal-directed behavior and peripheral nutrient partitioning.

## AgRP NEURONS ARE NECESSARY AND SUFFICIENT TO INITIATE THE FULL FEEDING SEQUENCE

In 2005, several laboratories reported the selective ablation of AgRP neurons. Although the methods and the results differed somewhat, there was agreement that acute depletion of AgRP neurons in the adult mouse leads to life-threatening anorexia (Bewick et al., 2005; Gropp et al., 2005; Luquet et al., 2005; Xu et al., 2005). These experiments demonstrated that ablation of AgRP neurons in adult mice inhibits feeding and can lead to starvation. Ablation of AgRP neurons still caused severe anorexia when performed in the genetic context of A^y^ mice (Wu et al., 2008a), a model in which the melanocortin signaling pathway is already tonically inhibited by the ectopic expression of the melanocortin receptor antagonist, agouti (Miltenberger et al., 1997). These data indicated that the anorexia is not the direct consequence of unopposed melanocortin tone. Wu et al. (2008a) went on to show that acute loss of GABA signaling by AgRP neurons was responsible. Although ablation of AgRP neurons in adult mice leads to starvation, mice can adapt to the loss of AgRP neurons and continue to eat adequately. This was first shown by performing the ablation in neonatal mice, before AgRP neurons are mature (Luquet et al., 2005), but it was subsequently shown that this phenomenon can also occur in adult mice (Wu et al., 2009, 2012a,b).

Direct activation of AgRP neurons *in vivo *has been achieved through either forced expression of designer receptors exclusively activated by designer drugs (DREADD) or photoactivated channel rhodopsin allowing for chemical- or light-mediated activation of neurons (Hegemann and Moglich, 2011; Krashes et al., 2011). Using optogenetic techniques, Aponte et al. (2011) found that photoactivation of AgRP neurons promoted feeding in both wild-type and A^y^ mice. In a subsequent study, the Sternson group showed that photoactivation of feeding is mediated in part by activation of OT-expressing neurons in the PVN (Atasoy et al., 2012). They also confirmed that inhibition of POMC neurons is neither necessary nor sufficient to trigger feeding since co-stimulation of both POMC and AgRP neurons resulted in rapid feeding response. These results provide an entirely new perspective to the field by showing that extinction of α-MSH signaling cascade was not mandatory for AgRP neurons to initiate feeding.

## PROCESSING OF TASTE AND VISCEROSENSORY INPUTS IN THE HYPOTHALAMUS–PONS–MEDULLA AXIS

**FIGURE 2:**
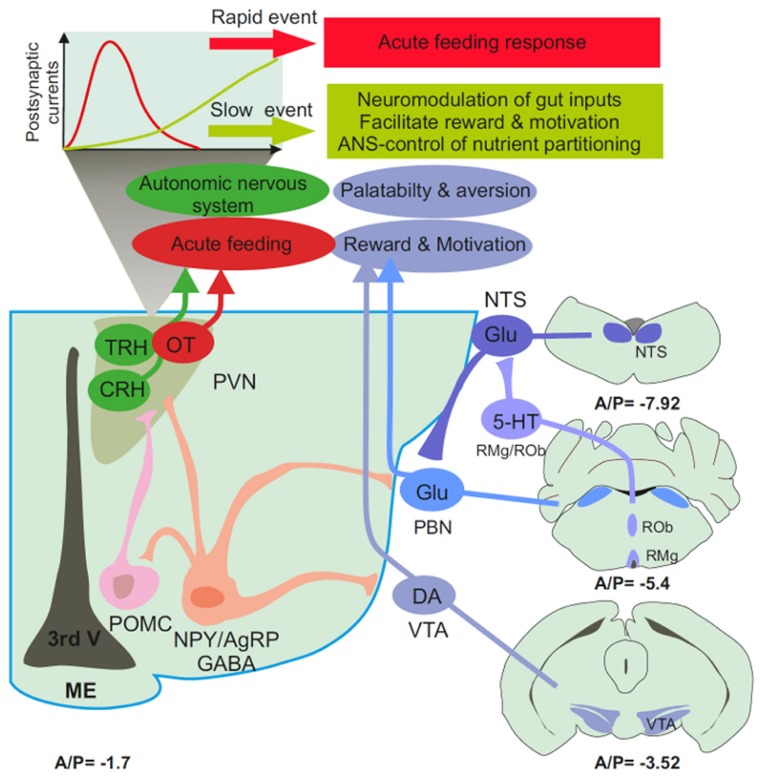
**Schematic representation of the feeding-neural circuitry**. Arcuate AgRP neurons are located at the bottom of the third ventricle (3rd V) close to the circumventricular organs, the median eminence (ME). They exert a GABAergic tonic inhibition onto POMC-, OT-, CRH-, and TRH-expressing neurons from the PVN, the PBN and the dopaminergic neurons (DA) of the VTA. The PBN receives glutamatergic input from the NTS which is also a target for serotoninergic neurons of the raphe obscurus (ROb) and the raphe magnus (RMg). In the PVN, the synaptic properties of AgRP axons are such that GABA release could promote post-synaptic inhibition through the long after the propagation of action potential. The timescale of this electrical event is compatible with the neuromodulation of post-synaptic targets including the VTA, the PBN, and preganglionic structure of the PVN. Hence, hunger-activated neurons could have a role that extends beyond the acute regulation of feeding to autonomic control of nutrient partitioning, the modulation of gut-borne signals in the brainstem, and the fine tuning mesolimbic reward and motivational circuitry. (A/P) Antero-posterior stereotaxic coordinates are presented in mm from bregma below each section.

By using Fos immunostaining to reveal neuron activation, Wu et al. (2008b) found that acute ablation of AgRP neurons in the PBN induces hyperactivity in all known targets of axonal projections from AgRP neurons. Moreover, they showed that GABA replacement in the PBN prevented anorexia and body weight loss after AgRP neuron ablation. This study demonstrated that GABA made by AgRP neurons was critically required to mediate their action in a melanocortin-independent manner (Wu et al., 2009; Wu and Palmiter, 2011) and put a new light on the PBN, a pontine structure that links gustatory sensory circuits to the brain center that processes the reward and motivational aspects of feeding (de Araujo, 2009; Suwabe and Bradley, 2009; Tokita et al., 2009; Oliveira-Maia et al., 2011). Gut-initiated viscerosensitive satiety or aversive signals together with food-related cues gathered by sensory neurons innervating the oral cavity are routed to the NTS primarily by the afferent portion of the vagus nerve (Schwartz et al., 1991, 1993; Moran et al., 2001; Schwartz and Moran, 2002). In the rodent, the PBN is a second-order target for NTS taste-related information. It serves as a relay structure for the encoding of the reward and motivational components of food-associated cues through the activation of the mesolimbic dopaminergic system (de Araujo, 2009; Suwabe and Bradley, 2009; Tokita et al., 2009; Oliveira-Maia et al., 2011). Looking for the source of excitatory inputs into the PBN that mediate its hyperactivity once the GABAergic tone from AgRP neurons is removed, Wu et al. (2012a) demonstrated that input to the PBN is glutamatergic and that it arises from a subpopulation of viscerosensitive NTS neurons. They also showed that the latter received tonic activation from serotoninergic neurons of the raphe obscurus and the raphe magnus (**Figures 1 and 2**).

The rostral NTS is also a target of descending projections from cognitive and emotional processing centers such as the insular and prefrontal cortex, the central nucleus of the amygdala, the lateral hypothalamus, and the BNST. The study from Wu et al. (2012a) provides an additional dimension to the hypothalamus–pons–medulla axis connection by suggesting a direct role for AgRP neurons in the ability of the medulla to relay rewarding and aversive information and integrate cognitive and emotional feedback from limbic structures (**Figure 1**). This Arc–pons–medulla axis could be instrumental in the gain of body weight that is often associated with the treatment of depression of bipolar disorders using selective serotonin re-uptake inhibitors.

Based on the circuitry described, one would expect that activation of AgRP neurons stimulate feeding by activating OT-expressing neurons in the PVN, while at the same time dampening activation of the PBN and thereby minimizing the influence of satiety and or visceral malaise. Photoactivation of AgRP axons in the PBN did not promote feeding, whereas activation of the PVN resulted in robust feeding (Atasoy et al., 2012). However, reducing satiety or visceral malaise would not necessarily promote robust feeding. Thus, the discrepancy may be resolved by considering the timescale at which the two events occur as well as their potential contribution to body weight maintenance. AgRP input to the PVN could convey an acute hunger-activated feeding response whereas AgRP input to the PBN could be more tonic in nature and involve longer-lasting actions that insure the proper excitatory balance of the PBN.

## CONNECTING METABOLIC NEEDS AND GOAL-DIRECTED BEHAVIOR

A recent study provides evidence that AgRP neurons could directly participate in the dopaminergic encoding of goal-directed behavior independent from the actual retrieval of food. The reinforcing and motivational aspects of food are closely tied to the release of the neurotransmitter dopamine by midbrain dopamine neurons in the ventral tegmental area (VTA) that project to the nucleus accumbens, and other limbic brain regions. VTA-mediated dopamine release is stimulated by high-fat/high-sugar foods as well as by most other objects of desire (e.g., sex, drugs; Wise, 2006). The VTA–striatal network provides a crucial neural substrate upon which drugs of abuse (e.g., cocaine, nicotine, morphine) exert their effect; thus, this projection is often referred to as the brain “reward circuit” (Kelley et al., 2005).

Using a model in which AgRP neurons are either ablated from birth or rendered hypoactive through selective knockdown of the metabolic sensor of the sirtuin family Sirt1 (silent mating type information regulation 2 homolog), a direct role for AgRP neurons in modulation of dopamine signaling was revealed (Dietrich et al., 2012). They demonstrated a direct projection from AgRP neurons to the VTA and inactivation or ablation of AgRP neurons reduced GABAergic tone to VTA dopamine neurons. This translated to higher excitability of VTA neurons and facilitated the induction of long-term potentiation. Hence, a reduced activity of AgRP neurons resulted in enhanced dopamine-dependent encoding of reward and motivation. At the behavioral level, the two models showed an enhanced response to novelty and a stronger preference for an environment associated with cocaine injection (Dietrich et al., 2012; Palmiter, 2012).

One can therefore envision GABAergic tone from AgRP neurons to the PBN and the VTA as a necessary input to counteract gut-borne aversive input while reducing the threshold at which taste-related information is successfully transferred to cognitive, emotional, and rewarding processing centers in the brain (**Figure 1**). This mechanism could be central to the maintenance of the motivational and rewarding components of feeding when energy stores are low and when the food is deprived of reinforcing properties. The activity of AgRP neurons would be necessary to maintain metabolic need as a significant contributor of goal-directed behavior. Decreased AgRP neuronal input, tonic or phasic, could result in the progressive replacement of hunger by limbic-associated emotional inputs such as stress or anxiety. The overall consequence could be that a drive for reward seeking eventually prevails over metabolic demand in the control of feeding (Dallman et al., 2005; **Figure 2**).

## ORCHESTRATING NUTRIENT PARTITIONING THROUGH TONIC CONTROL OF PREGANGLIONIC STRUCTURES

Agouti-related protein neurons project to several preganglionic structures (Broberger et al., 1998) including the regions involved in activating autonomic nervous system (ANS) output such as the PVN (Cowley et al., 1999). ANS innervation of peripheral tissues (pancreas, liver, visceral adipose tissue) has a distinct organization that can be tracked back to pre-autonomic hypothalamic neurons (Kreier et al., 2006). Nutrient partitioning (the integrated processes that control conversion, storage, and utilization of nutrients) relies on the ability of the brain to orchestrate peripheral organ activity through the modulation of the ANS. Using a model of neonatal depletion, we showed that the lack of AgRP neurons affects the relative balance between lipid and carbohydrate metabolism. As a consequence, mice lacking AgRP neurons became obese and hyperinsulinemic on regular chow but displayed reduced body weight gain and paradoxical improvement in glucose tolerance on high-fat diet (Joly-Amado et al., 2012). This action was independent from feeding, and involved GABAergic input to preganglionic structures and the consequent modulation of ANS output onto liver, muscle, and pancreas.

These findings indicate that AgRP neurons play a role that extends beyond the regulation of feeding, to the control of peripheral nutrient partitioning. In good agreement, a recent study showed that Sirt 1 inactivation selectively in AgRP neurons resulted in a shift in the overall metabolic profile, the impairment of metabolic adaptation induced by a fast, and a change in ghrelin-induced excitability of AgRP neurons (Dietrich et al., 2010). Importantly, we found that a GABA_A_ receptor agonist normalized the metabolic profile in mice lacking AgRP neurons (Joly-Amado et al., 2012). These results suggest that, here too, GABA may be a crucial signaling molecule by which AgRP neurons control peripheral nutrient partitioning.

In conclusion, several recent studies have significantly broadened the spectrum of brain structure, mechanism, and timescale that underlie the action of AgRP neurons in the regulation of energy balance. The intrinsic nature of GABA release has received much attention and technological tools and approaches have allowed dissociation of the behavioral and metabolic actions of AgRP from the melanocortin signaling pathway. Long-lasting inhibitory currents that persist long after the action potential indicate a putative function for AgRP neurons in the neuromodulation of post-synaptic targets. A second era is just beginning for this neurocircuitry that will likely provide fundamental insights into the mechanisms that underlie not only food-related but also non-food-related behavior including cognitive, emotional, and reward processes.

## Conflict of Interest Statement

The authors declare that the research was conducted in the absence of any commercial or financial relationships that could be construed as a potential conflict of interest.

## References

[B1] Aponte Y. Atasoy D. Sternson S. M. (2011). AGRP neurons are sufficient to orchestrate feeding behavior rapidly and without training. *Nat. Neurosci.* 14 351–355 21209617 10.1038/nn.2739 PMC3049940

[B2] Atasoy D. Betley J. N. Su H. H. Sternson S. M. (2012). Deconstruction of a neural circuit for hunger. *Nature* 488 172–177 22801496 10.1038/nature11270 PMC3416931

[B3] Bewick G. A. Gardiner J. V. Dhillo W. S. Kent A. S. White N. E. Webster Z. (2005). Post-embryonic ablation of AgRP neurons in mice leads to a lean, hypophagic phenotype. *FASEB J.* 19 1680–1682 16099943 10.1096/fj.04-3434fje

[B4] Broberger C. Hokfelt T. (2001). Hypothalamic and vagal neuropeptide circuitries regulating food intake. *Physiol. Behav.* 74 669–682 11790430 10.1016/s0031-9384(01)00611-4

[B5] Broberger C. Johansen J. Johansson C. Schalling M Hokfelt T. (1998). The neuropeptide Y/agouti gene-related protein (AGRP) brain circuitry in normal, anorectic, and monosodium glutamate-treated mice. *Proc. Natl. Acad. Sci. U.S.A.* 95 15043–15048 9844012 10.1073/pnas.95.25.15043 PMC24572

[B6] Clark J. T. Kalra P. S. Crowley W. R. Kalra S. P. (1984). Neuropeptide Y and human pancreatic polypeptide stimulate feeding behavior in rats. *Endocrinology* 115 427–429 6547387 10.1210/endo-115-1-427

[B7] Cone R. D. (2005). Anatomy and regulation of the central melanocortin system. *Nat. Neurosci.* 8 571–578 15856065 10.1038/nn1455

[B8] Cowley M. A. Pronchuk N. Fan W. Dinulescu D. M. Colmers W. F. Cone R. D. (1999). Integration of NPY, AGRP, and melanocortin signals in the hypothalamic paraventricular nucleus: evidence of a cellular basis for the adipostat. *Neuron* 24 155–163 10677034 10.1016/s0896-6273(00)80829-6

[B9] Cowley M. A. Smart J. L. Rubinstein M. Cerdan M. G. Diano S. Horvath T. L. (2001). Leptin activates anorexigenic POMC neurons through a neural network in the arcuate nucleus. *Nature* 411 480–484 11373681 10.1038/35078085

[B10] Dallman M. F. Pecoraro N. C La Fleur S. E. (2005). Chronic stress and comfort foods: self-medication and abdominal obesity. *Brain Behav. Immun.* 19 275–280 15944067 10.1016/j.bbi.2004.11.004

[B11] de Araujo I. E. (2009). Gustatory and homeostatic functions of the rodent parabrachial nucleus. *Ann. N. Y. Acad. Sci.* 1170 383–391 19686163 10.1111/j.1749-6632.2009.03923.x

[B12] Dietrich M. O. Antunes C. Geliang G. Liu Z. W. Borok E. Nie Y. (2010). Agrp neurons mediate Sirt1’s action on the melanocortin system and energy balance: roles for Sirt1 in neuronal firing and synaptic plasticity. *J. Neurosci.* 30 11815–11825 20810901 10.1523/JNEUROSCI.2234-10.2010 PMC2965459

[B13] Dietrich M. O. Bober J. Ferreira J. G. Tellez L. A. Mineur Y. S. Souza D. O. (2012). AgRP neurons regulate development of dopamine neuronal plasticity and nonfood-associated behaviors. *Nat. Neurosci.* 15 1108–1110 22729177 10.1038/nn.3147 PMC3411867

[B14] Fan W. Boston B. A. Kesterson R. A. Hruby V. J. Cone R. D. (1997). Role of melanocortinergic neurons in feeding and the agouti obesity syndrome. *Nature* 385 165–168 8990120 10.1038/385165a0

[B15] Fekete C. Legradi G. Mihaly E. Huang Q. H. Tatro J. B. Rand W. M. (2000). alpha-Melanocyte-stimulating hormone is contained in nerve terminals innervating thyrotropin-releasing hormone-synthesizing neurons in the hypothalamic paraventricular nucleus and prevents fasting-induced suppression of prothyrotropin-releasing hormone gene expression. *J. Neurosci.* 20 1550–1558 10662844 10.1523/JNEUROSCI.20-04-01550.2000 PMC6772359

[B16] Flier J. S. (2006). AgRP in energy balance: will the real AgRP please stand up? *Cell Metab.* 3 83–85 16459309 10.1016/j.cmet.2006.01.003

[B17] Fu L. Y van den Pol A. N. (2008). Agouti-related peptide and MC3/4 receptor agonists both inhibit excitatory hypothalamic ventromedial nucleus neurons. *J. Neurosci.* 28 5433–5449 18495877 10.1523/JNEUROSCI.0749-08.2008 PMC2634774

[B18] Gropp E. Shanabrough M. Borok E. Xu A. W. Janoschek R. Buch T. (2005). Agouti-related peptide-expressing neurons are mandatory for feeding. *Nat. Neurosci.* 8 1289–1291 16158063 10.1038/nn1548

[B19] Hahn T. M. Breininger J. F. Baskin D. G. Schwartz M. W. (1998). Coexpression of Agrp and NPY in fasting-activated hypothalamic neurons. *Nat. Neurosci.* 1 271–272 10195157 10.1038/1082

[B20] Haskell-Luevano C. Chen P. Li C. Chang K. Smith M. S. Cameron J. L. (1999). Characterization of the neuroanatomical distribution of agouti-related protein immunoreactivity in the rhesus monkey and the rat. *Endocrinology* 140 1408–1415 10067869 10.1210/endo.140.3.6544

[B21] Haskell-Luevano C. Monck E. K. (2001). Agouti-related protein functions as an inverse agonist at a constitutively active brain melanocortin-4 receptor. *Regul Pept.* 99 1–7 11257308 10.1016/s0167-0115(01)00234-8

[B22] Hegemann P. Moglich A. (2011). Channelrhodopsin engineering and exploration of new optogenetic tools. *Nat. Methods* 8 39–42 21191371 10.1038/nmeth.f.327

[B23] Hinney A. Schmidt A. Nottebom K. Heibult O. Becker I. Ziegler A. (1999). Several mutations in the melanocortin-4 receptor gene including a nonsense and a frameshift mutation associated with dominantly inherited obesity in humans. *J. Clin. Endocrinol. Metab.* 84 1483–1486 10199800 10.1210/jcem.84.4.5728

[B24] Horvath T. L. Bechmann I. Naftolin F. Kalra S. P. Leranth C. (1997). Heterogeneity in the neuropeptide Y-containing neurons of the rat arcuate nucleus: GABAergic and non-GABAergic subpopulations. *Brain Res.* 756 283–286 9187344 10.1016/s0006-8993(97)00184-4

[B25] Horvath T. L. Naftolin F. Kalra S. P. Leranth C. (1992). Neuropeptide-Y innervation of beta-endorphin-containing cells in the rat mediobasal hypothalamus: a light and electron microscopic double immunostaining analysis. *Endocrinology* 131 2461–2467 1425443 10.1210/endo.131.5.1425443

[B26] Huszar D. Lynch C. A. Fairchild-Huntress V. Dunmore J. H. Fang Q. Berkemeier L. R. (1997). Targeted disruption of the melanocortin-4 receptor results in obesity in mice. *Cell* 88 131–141 9019399 10.1016/s0092-8674(00)81865-6

[B27] Jegou S. Boutelet I. Vaudry H. (2000). Melanocortin-3 receptor mRNA expression in pro-opiomelanocortin neurones of the rat arcuate nucleus. *J. Neuroendocrinol.* 12 501–505 10844578 10.1046/j.1365-2826.2000.00477.x

[B28] Joly-Amado A. Denis R. G. Castel J. Lacombe A. Cansell C. Rouch C. (2012). Hypothalamic AgRP-neurons control peripheral substrate utilization and nutrient partitioning. *EMBO J.* 31 4276–4288 22990237 10.1038/emboj.2012.250 PMC3501217

[B29] Kelley A. E. Baldo B. A. Pratt W. E. (2005). A proposed hypothalamic-thalamic-striatal axis for the integration of energy balance, arousal, and food reward. *J. Comp. Neurol.* 493 72–85 16255002 10.1002/cne.20769

[B30] Krashes M. J. Koda S. Ye C. Rogan S. C. Adams A. C. Cusher D. S. (2011). Rapid, reversible activation of AgRP neurons drives feeding behavior in mice. *J. Clin. Invest.* 121 1424–1428 21364278 10.1172/JCI46229 PMC3069789

[B31] Kreier F. Kap Y. S. Mettenleiter T. C. Van Heijningen C. Van Der Vliet J. Kalsbeek A. (2006). Tracing from fat tissue, liver, and pancreas: a neuroanatomical framework for the role of the brain in type 2 diabetes. *Endocrinology* 147 1140–1147 16339209 10.1210/en.2005-0667

[B32] Krude H. Schnabel D. Luck W. Gruters A. (1999). Implications of the phenotype of POMC deficiency for the role of POMC-derived peptides in skin physiology. *Ann. N. Y. Acad. Sci.* 885 419–421 10816678 10.1111/j.1749-6632.1999.tb08702.x

[B33] Lechan R. M. Fekete C. (2006). The TRH neuron: a hypothalamic integrator of energy metabolism. *Prog. Brain Res.* 153 209–235 16876577 10.1016/S0079-6123(06)53012-2

[B34] Lein E. S. Hawrylycz M. J. Ao N. Ayres M. Bensinger A. Bernard A. (2007). Genome-wide atlas of gene expression in the adult mouse brain. *Nature* 445 168–176 17151600 10.1038/nature05453

[B35] Liu H. Kishi T. Roseberry A. G. Cai X. Lee C. E. Montez J. M. (2003). Transgenic mice expressing green fluorescent protein under the control of the melanocortin-4 receptor promoter. *J. Neurosci.* 23 7143–7154 12904474 10.1523/JNEUROSCI.23-18-07143.2003 PMC6740648

[B36] Lu X. Y. Barsh G. S. Akil H. Watson S. J. (2003). Interaction between alpha-melanocyte-stimulating hormone and corticotropin-releasing hormone in the regulation of feeding and hypothalamo-pituitary-adrenal responses. *J. Neurosci.* 23 7863–7872 12944516 10.1523/JNEUROSCI.23-21-07863.2003 PMC6740604

[B37] Luquet S. Perez F. A. Hnasko T. S. Palmiter R. D. (2005). NPY/AgRP neurons are essential for feeding in adult mice but can be ablated in neonates. *Science* 310 683–685 16254186 10.1126/science.1115524

[B38] Michaud E. J. Bultman S. J. Klebig M. L. Van Vugt M. J. Stubbs L. J. Russell L. B. (1994). A molecular model for the genetic and phenotypic characteristics of the mouse lethal yellow (Ay) mutation. *Proc. Natl. Acad. Sci. U.S.A.* 91 2562–2566 8146154 10.1073/pnas.91.7.2562 PMC43409

[B39] Miltenberger R. J. Mynatt R. L. Wilkinson J. E. Woychik R. P. (1997). The role of the agouti gene in the yellow obese syndrome. *J. Nutr.* 127 1902S–1907S 9278579 10.1093/jn/127.9.1902S

[B40] Moran T. H. Ladenheim E. E. Schwartz G. J. (2001). Within-meal gut feedback signaling. *Int. J. Obes. Relat. Metab. Disord.* 25(Suppl. 5) S39–S41 11840213 10.1038/sj.ijo.0801910

[B41] Mountjoy K. G. Wong J. (1997). Obesity, diabetes and functions for proopiomelanocortin-derived peptides. *Mol. Cell. Endocrinol.* 128 171–177 9140088 10.1016/s0303-7207(96)04017-8

[B42] Nijenhuis W. A. Oosterom J. Adan R. A. (2001). AgRP(83-132) acts as an inverse agonist on the human-melanocortin-4 receptor. *Mol. Endocrinol.* 15 164–171 11145747 10.1210/mend.15.1.0578

[B43] Oliveira-Maia A. J. Roberts C. D. Simon S. A. Nicolelis M. A. (2011). Gustatory and reward brain circuits in the control of food intake. *Adv. Tech. Stand. Neurosurg.* 36 31–59 21197607 10.1007/978-3-7091-0179-7_3 PMC3434955

[B44] Ollmann M. M. Wilson B. D. Yang Y. K. Kerns J. A. Chen Y. Gantz I. (1997). Antagonism of central melanocortin receptors in vitro and in vivo by agouti-related protein. *Science* 278 135–138 9311920 10.1126/science.278.5335.135

[B45] Palmiter R. D. (2012). New game for hunger neurons. *Nat. Neurosci.* 15 1060–1061 22837033 10.1038/nn.3167

[B46] Pinto S. Roseberry A. G. Liu H. Diano S. Shanabrough M. Cai X. (2004). Rapid rewiring of arcuate nucleus feeding circuits by leptin. *Science* 304 110–115 15064421 10.1126/science.1089459

[B47] Schwartz G. J. Mchugh P. R. Moran T. H. (1991). Integration of vagal afferent responses to gastric loads and cholecystokinin in rats. *Am. J. Physiol.* 261 R64–R69 1858957 10.1152/ajpregu.1991.261.1.R64

[B48] Schwartz G. J. Mchugh P. R. Moran T. H. (1993). Gastric loads and cholecystokinin synergistically stimulate rat gastric vagal afferents. *Am. J. Physiol.* 265 R872–R876 8238459 10.1152/ajpregu.1993.265.4.R872

[B49] Schwartz G. J. Moran T. H. (2002). Leptin and neuropeptide y have opposing modulatory effects on nucleus of the solitary tract neurophysiological responses to gastric loads: implications for the control of food intake. *Endocrinology* 143 3779–3784 12239088 10.1210/en.2002-220352

[B50] Shutter J. R. Graham M. Kinsey A. C. Scully S. Luthy R. Stark K. L. (1997). Hypothalamic expression of ART, a novel gene related to agouti, is up-regulated in obese and diabetic mutant mice. *Genes Dev.* 11 593–602 9119224 10.1101/gad.11.5.593

[B51] Suwabe T. Bradley R. M. (2009). Characteristics of rostral solitary tract nucleus neurons with identified afferent connections that project to the parabrachial nucleus in rats. *J. Neurophysiol.* 102 546–555 19439671 10.1152/jn.91182.2008 PMC2712278

[B52] Tatemoto K. Carlquist M. Mutt V. (1982). Neuropeptide Y – a novel brain peptide with structural similarities to peptide YY and pancreatic polypeptide. *Nature* 296 659–660 6896083 10.1038/296659a0

[B53] Tokita K. Inoue T. Boughter J. D. Jr (2009). Afferent connections of the parabrachial nucleus in C57BL/6J mice. *Neuroscience* 161 475–488 19327389 10.1016/j.neuroscience.2009.03.046 PMC2705209

[B54] Tolle V. Low M. J. (2008). In vivo evidence for inverse agonism of Agouti-related peptide in the central nervous system of proopiomelanocortin-deficient mice. *Diabetes* 57 86–94 17909095 10.2337/db07-0733

[B55] Williams G. Bing C. Cai X. J. Harrold J. A. King P. J. Liu X. H. (2001). The hypothalamus and the control of energy homeostasis: different circuits, different purposes. *Physiol. Behav.* 74 683–701 11790431 10.1016/s0031-9384(01)00612-6

[B56] Wise R. A. (2006). Role of brain dopamine in food reward and reinforcement. *Philos. Trans. R. Soc. Lond. B Biol. Sci.* 361 1149–1158 16874930 10.1098/rstb.2006.1854 PMC1642703

[B57] Wu Q. Boyle M. P. Palmiter R. D. (2009). Loss of GABAergic signaling by AgRP neurons to the parabrachial nucleus leads to starvation. *Cell* 137 1225–1234 19563755 10.1016/j.cell.2009.04.022 PMC2729323

[B58] Wu Q. Clark M. S. Palmiter R. D. (2012a). Deciphering a neuronal circuit that mediates appetite. *Nature* 483 594–597 22419158 10.1038/nature10899 PMC4000532

[B59] Wu Q. Whiddon B. B. Palmiter R. D. (2012b). Ablation of neurons expressing agouti-related protein, but not melanin concentrating hormone, in leptin-deficient mice restores metabolic functions and fertility. *Proc. Natl. Acad. Sci. U.S.A.* 109 3155–3160 22232663 10.1073/pnas.1120501109 PMC3286929

[B60] Wu Q. Howell M. P. Cowley M. A. Palmiter R. D. (2008a). Starvation after AgRP neuron ablation is independent of melanocortin signaling. *Proc. Natl. Acad. Sci. U.S.A.* 105 2687–2692 18272480 10.1073/pnas.0712062105 PMC2268197

[B61] Wu Q. Howell M. P. Palmiter R. D. (2008b). Ablation of neurons expressing agouti-related protein activates fos and gliosis in postsynaptic target regions. *J. Neurosci.* 28 9218–9226 18784302 10.1523/JNEUROSCI.2449-08.2008 PMC2597113

[B62] Wu Q. Palmiter R. D. (2011). GABAergic signaling by AgRP neurons prevents anorexia via a melanocortin-independent mechanism. *Eur. J. Pharmacol.* 660 21–27 21211531 10.1016/j.ejphar.2010.10.110 PMC3108334

[B63] Xu A. W. Kaelin C. B. Morton G. J. Ogimoto K. Stanhope K. Graham J. (2005). Effects of hypothalamic neurodegeneration on energy balance. *PLoS Biol.* 3:e415 10.1371/journal.pbio.0030415 PMC1287504 16296893

